# Development and validation of a unifying pre-treatment decision tool for intracranial and extracranial metastasis-directed radiotherapy

**DOI:** 10.3389/fonc.2023.1095170

**Published:** 2023-03-27

**Authors:** Roman Kowalchuk, Trey C. Mullikin, William Breen, Hunter C. Gits, Marcus Florez, Brian De, William S. Harmsen, Peter Sean Rose, Brittany L. Siontis, Brian A. Costello, Jonathan M. Morris, John J. Lucido, Kenneth R. Olivier, Brad Stish, Nadia N. Laack, Sean Park, Dawn Owen, Amol J. Ghia, Paul D. Brown, Kenneth Wing Merrell

**Affiliations:** ^1^ Department of Radiation Oncology, Mayo Clinic, Rochester, MN, United States; ^2^ Department of Radiation Oncology, Duke University, Durham, NC, United States; ^3^ Department of Radiation Oncology, Houston, MD Anderson Cancer Center, Houston, TX, United States; ^4^ Mayo Clinic, Department of Statistics, Rochester, MN, United States; ^5^ Mayo Clinic, Department of Orthopedic Surgery, Rochester, MN, United States; ^6^ Mayo Clinic, Department of Radiology, Rochester, MN, United States; ^7^ Mayo Clinic, Department of Medical Oncology, Rochester, MN, United States; ^8^ Mayo Clinic, Department of Medical Physics, Rochester, MN, United States

**Keywords:** metastatic disease, metastasis-directed radiotherapy, oligometastasis, modeling, outcomes

## Abstract

**Background:**

Though metastasis-directed therapy (MDT) has the potential to improve overall survival (OS), appropriate patient selection remains challenging. We aimed to develop a model predictive of OS to refine patient selection for clinical trials and MDT.

**Patients and methods:**

We assembled a multi-institutional cohort of patients treated with MDT (stereotactic body radiation therapy, radiosurgery, and whole brain radiation therapy). Candidate variables for recursive partitioning analysis were selected per prior studies: ECOG performance status, time from primary diagnosis, number of additional non-target organ systems involved (NOS), and intracranial metastases.

**Results:**

A database of 1,362 patients was assembled with 424 intracranial, 352 lung, and 607 spinal treatments (n=1,383). Treatments were split into training (TC) (70%, n=968) and internal validation (IVC) (30%, n=415) cohorts. The TC had median ECOG of 0 (interquartile range [IQR]: 0-1), NOS of 1 (IQR: 0-1), and OS of 18 months (IQR: 7-35). The resulting model components and weights were: ECOG = 0, 1, and > 1 (0, 1, and 2); 0, 1, and > 1 NOS (0, 1, and 2); and intracranial target (2), with lower scores indicating more favorable OS. The model demonstrated high concordance in the TC (0.72) and IVC (0.72). The score also demonstrated high concordance for each target site (spine, brain, and lung).

**Conclusion:**

This pre-treatment decision tool represents a unifying model for both intracranial and extracranial disease and identifies patients with the longest survival after MDT who may benefit most from aggressive local therapy. Carefully selected patients may benefit from MDT even in the presence of intracranial disease, and this model may help guide patient selection for MDT.

## Introduction

Recent studies have explored oligometastatic disease, the idea that select patients with limited metastatic disease burden may benefit from metastasis-directed therapy (MDT) (1). MDT typically includes interventional radiology techniques, surgical resection, and radiotherapy, often using stereotactic planning techniques involving a sharp dose fall-off to a focal target (2). Clinical trials are ongoing to optimize patient selection for MDT to maximize cost-effectiveness, quality of life, and survival while minimizing side effects of treatment (3–6). The SABR-COMET trial randomized 99 patients with one to five metastatic sites to standard of care with or without stereotactic body radiation therapy (SBRT) to each of the metastatic lesions, demonstrating an improvement in overall survival (OS) with SBRT (2, 7–9). The ORIOLE and STOMP trials specifically assessed patients with primary prostate cancer and up to 3 sites of metastatic disease, and MDT improved 6 month progression-free and androgen deprivation therapy-free survival (10, 11). These encouraging results have led to the design of numerous other clinical trials studying MDT, including NRG-BR002 and SABR-COMET-10 (12, 13).

Despite differences in inclusion of primary histologies and type of MDT delivered, most clinical trials share the same fundamental patient inclusion criteria. First, the majority of clinical trials evaluating outcomes in oligometastatic cancer include patients with 5 or fewer metastatic lesions, with the assumption that the number of lesions, not the number of organs involved, is the ideal stratification. Second, clinical trials commonly exclude patients with intracranial disease based on the assumption that patients with brain metastases have a poor prognosis (14). However, recent analyses have questioned these assumptions and stratification factors, with emerging evidence that the number of organ systems involved by metastatic disease may better predict OS than the number of individual metastatic lesions (15–18). Furthermore, prognostication by the number of metastatic lesions is challenging in the setting of new imaging modalities that identify previously occult sites of disease, whose influence on a patient’s prognosis remains to be clearly defined (19). Some patients treated for brain metastasis may also have durable OS similar to metastatic disease to other organs, and applicability of intracranial-specific models to other sites of MDT (e.g. spine SBRT) is yet untested (20, 21). Substantial work involving the generation of high-fidelity prognostic models has been pioneered by Sperduto et al., and our group aims to extend this work regarding intracranial disease by focusing on extracranial disease (20).

Given the clinical uncertainty of the ideal patient selection for aggressive MDT, we analyzed a large, multi-institutional database of patients treated with MDT involving the brain, lungs, or spine to develop and validate a unifying prognostic model applicable for both intracranial and extracranial disease to guide treatment selection and future clinical trial design.

## Methods

### Patient cohort

The dataset was comprised of 4 separate databases from 2 large academic centers. The first institution provided 3 cohorts of patients who received MDT (1): brain, (2) lung, and (3) spine metastases. The second institution contributed a single cohort of patients who received MDT for spine metastases. More detailed information concerning each underlying dataset is described in the corresponding publications, as well as in **Supplemental Table 1** (17, 22, 23). MDT for brain metastases included whole brain radiation therapy (WBRT) and/or stereotactic radiosurgery (SRS) (24). This decision was made to allow for comparison to the prognostic models generated by Sperduto et al. (20). Furthermore, given the lack of consensus concerning the threshold (either volumetric or number of metastatic lesions) for recommendation of WBRT and SRS, both were considered within the analysis. MDT for spine and lung metastasis included only SBRT. Treatments with conventional palliative dose-fractionation schemes for spine and lung targets were excluded. The decision to treat a patient as having “oligometastatic disease” was defined per the clinical discretion of the treating multi-disciplinary team, as there is no validated consensus definition. Decisions regarding treating all sites of disease with MDT or alternative approaches (e.g. treatment of only a dominant lesion) were also left to the discretion of the treating physicians, secondary to a lack of consensus clinical standard of practice. Institutional review board (IRB) approval was obtained at both institutions. Benign primary tumors were excluded. If a patient had multiple treatments targeting the same organ (e.g. two spine SBRT treatments), only the first such treatment was included.

### Treatment

Treatment planning and delivery were conducted per institutional protocol. Rigid immobilization was utilized for stereotactic radiotherapy treatments, with narrow PTV margins of 0-5 mm. Motion management was considered for all cases of lung SBRT, including use of breath hold and phase gating, per the discretion of the treating physician. Stereotactic treatments were generally delivered using 1-5 daily treatments. Treatments of intracranial targets involved standard use of a 3-point mask for immobilization. Treatments with Gamma Knife were also conducted in accordance with institutional protocol. For WBRT, hippocampal avoidance and memantine were used whenever possible, with 10 daily fractions offered for most patients.

### Model generation

Candidate variable selection was determined per prior work involved the development of a prognostic model after SBRT for spinal metastases, with variable selection targeted for the primary endpoint of OS (17). This included thorough analysis of other potential prognostic factors, including but not limited to patient characteristics (e.g. age), local tumor characteristics (e.g. gross tumor volume), systemic therapy, and histology. After assessment of a wide range of candidate variables, the final model included: Eastern Cooperative Oncology Group (ECOG) performance status, number of non-target organ systems (NOS), and time from primary diagnosis (TPD). NOS was defined as the number of organ systems involved with disease outside of the organ system of the treatment target (i.e. bone for spine SBRT, lung for lung SBRT, and brain for intracranial treatments). Further, intracranial target was added as a fourth potential variable. The overall cohort was split into training (70%, n=968) and internal validation (30%, n=415) cohorts *via* a random number generator, and comparable patient characteristics were confirmed in both sets. Feature importance testing was conducted and a correlation heatmap was generated. The overarching training set was used for model generation and RPA analysis while the internal validation set was only used afterwards to validate the prognostic value of the resulting risk score.

Recursive partitioning analysis (RPA) was conducted with open-source packages in Python (version 3.8.0), with similar methodology to that reported in the literature (25–30). The overarching training set was used for all modeling efforts. RPA was conducted through decision-tree analysis using the overarching training set (70% of the overall cohort), split into training (60%), validation (20%), and test sets (20%). Potential RPA models were generated with the training and validation sets, and model performance was assessed *via* the independent test set (4). A minimum of 15 patients in each group was required for node splitting, but this criterion was analyzed as a range from 15-20 minimum patients. The log-rank test was used as the criterion for node splitting, as the primary endpoint of the analysis was OS. RPA trees were only allowed to contain a maximum of 4 groups to minimize overfitting. The highest-fidelity models were then considered, and the prognostic factors used in these models were considered for inclusion in the final risk score.

### Model validation

Additional statistical analyses were conducted with SAS version 9.4 (SAS Institute, Cary, NC). Cox proportional hazards analyses were conducted, and the resulting concordance value was reported as a measure of the fidelity of the resulting model and underlying variables. Such analyses were conducted for both the overarching training and internal validation sets to assess the prognostic value of the final risk score and individual component variables. Cox proportional hazards analyses were also performed for each distinct treatment target (brain, lung, and spine). Hazard ratios (HR) were reported, along with 95% confidence intervals (CI). For all analyses, p<0.05 was the threshold for statistical significance. Finally, the Kaplan-Meier method was used to plot OS as stratified by the resulting risk score.

## Results

### Patient cohort

A total of 1,260 individual patients were included with 424 brain, 352 lung, and 607 spinal targets (n=1,383). Further details concerning the patient demographics and primary histologies of each treatment target site are shown in **Supplemental Table 1**. For spine SBRT, 326 (54%) treatments involved single-fraction radiotherapy, and the median dose delivered for all fractionation schemes was 24 Gy (IQR: 20-30). For lung SBRT, a median of 5 fractions (IQR: 3-5) were used to deliver a median dose of 50 Gy (IQR: 50-54). Concerning intracranial treatments, 145 (34%) patients received initial WBRT, and 349 (82%) had initial SRS with or without WBRT. 78 patients (18%) had more than 10 brain metastases at the time of brain metastasis diagnosis, and of the remaining patients, a median 2 (IQR: 1-3) brain metastases were identified. Many patients with intracranial disease also had extracranial disease (75%), most commonly bone (43%) and lung (38%) lesions (**Supplemental Table 2**). The training set had median ECOG of 0 (IQR: 0-1), NOS of 1 (IQR: 0-1), and OS of 18 months (IQR: 7-35). The internal validation set demonstrated comparable characteristics to the training set (**Table 1**).

**Table 1:** Patient characteristics utilized for recursive partitioning analysis are demonstrated, as divided into training and validation sets.

	Training set (n=968)	Validation set (n=415)
Time from primary diagnosis (months, IQR)	35 (12-76)	37 (12-77)
Treatment target		
Intracranial	296 (31%)	128 (31%)
Lung	233 (24%)	119 (29%)
Spine	439 (45%)	168 (40%)
*Median ECOG (IQR)*	0 (0-1)	0 (0-1)
0	533 (55%)	221 (53%)
1	354 (37%)	155 (37%)
> 1	81 (8%)	39 (9%)
Median NOS (IQR)*	1 (0-1)	0 (0-1)
0	466 (48%)	223 (54%)
1	263 (27%)	90 (22%)
> 1	239 (25%)	102 (25%)
Overall survival (months, IQR)	18 (7-34)	19 (9-37)

IQR, interquartile range; GI, gastrointestinal; ECOG, Eastern Cooperative Oncology Group performance status; NOS, number of organ systems involved (outside of treatment target).

*NOS refers to the number of organ systems involved by disease outside of the organ system of the treatment target, per prior convention in the literature (15, 16).

### Risk score generation

**Figure 1:**
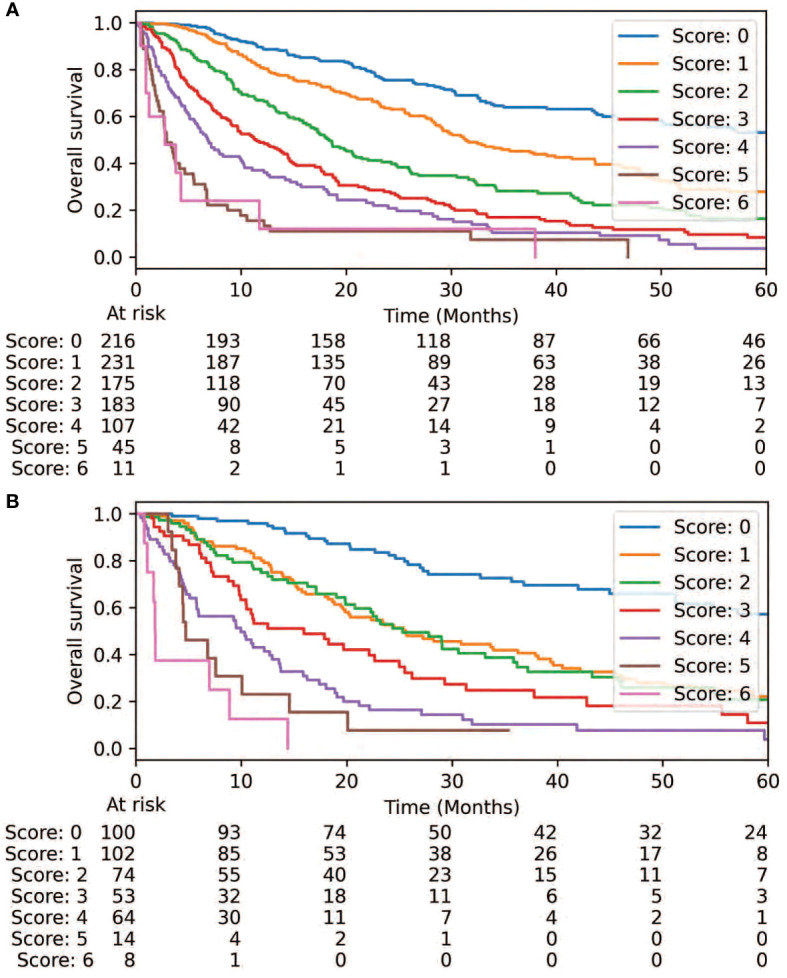
Overall survival is demonstrated *via* the prognostic score is demonstrated *via* the training set **(A)** and validation set **(B)**.

Feature importance testing failed to exclude any of the four candidate variables (ECOG, NOS, TPD, and intracranial) for consideration in the resulting risk score. A correlation heatmap also did not reveal substantial correlation between the variables, so all 4 were included as inputs into the RPA. Though ECOG, NOS, and intracranial were frequently included in the highest-fidelity models, TPD was not. It was, therefore, excluded from further consideration in the final model. Two separate thresholds for ECOG and NOS were identified *via* RPA: ECOG = 1 and > 1, and NOS = 1 and > 1. Cox proportional hazards analysis was performed and each of these thresholds demonstrated a statistically significant association with OS (**Table 2**). Corresponding weights were then approximated for development of the corresponding risk score: ECOG = 0, 1, and > 1 (0, 1, and 2, respectively); NOS = 0, 1, and > 1 (0, 1, and 2); and intracranial target (2) (**Table 3**). While ECOG > 1 had a higher hazard ratio (HR=4.22, 95% CI: 3.18-5.61, p<0.0001) than NOS > 1 (HR=2.12, 95% CI: 1.73-2.60, p<0.0001) and intracranial (HR=2.60, 95% CI: 2.16-3.13, p<0.0001), there were only 39 patients with ECOG > 1 in the internal validation set. For this reason, a higher relative weight for ECOG > 1 than NOS > 1 or intracranial was not assigned. The resulting risk score was plotted using the Kaplan-Meier method, revealing an association between the risk score and overall survival in the training and internal validation sets (**Figure 1**, p<0.0001). 2-year OS was 76%, 64%, 41%, 28%, 21%, 11%, and 12% for scores of 0, 1, 2, 3, 4, 5, and 6, respectively.

**Table 2:** The predictive power of the resulting model is demonstrated for the training and validation sets, with each individual model component also demonstrating statistical significance.

Model component	Training set (hazard ratio, 95% CI)	Validation set (hazard ratio, 95% CI)
ECOG		
0	1.0 (reference)	1.0 (reference)
1	1.82 (1.52-2.18), p<0.0001	1.94 (1.49-2.54), p<0.0001
> 1	4.22 (3.18-5.61), p<0.0001	5.73 (3.87-8.47), p<0.0001
NOS		
0	1.0 (reference)	1.0 (reference)
1	1.32 (1.07-1.62), p=0.01	1.75 (1.29-2.39), p=0.0004
> 1	2.12 (1.73-2.60), p<0.0001	2.48 (1.83-3.37), p<0.0001
*Intracranial*	2.60 (2.16-3.13), p<0.0001	2.03 (1.54-2.68), p<0.0001

CI, confidence interval; ECOG, Eastern Cooperative Oncology Group performance status; NOS, number of organ systems involved (outside of treatment target). Concordance values were 0.73 (95% confidence interval (CI): 0.71-0.75) and 0.72 (95% CI: 0.68-0.74) for the training and validation sets, respectively.

**Table 3:** The model components and their corresponding weights are tabulated.

Variable	Points
ECOG	+ 0 for ECOG = 0 + 1 for ECOG = 1 + 2 for ECOG > 1
NOS*	+ 0 for 0 organ systems + 1 for 1 organ system + 2 for > 1 organ systems
Intracranial target	+ 2

ECOG, Eastern Cooperative Oncology Group performance status; NOS, number of organ systems involved (outside of treatment target).

*NOS refers to the number of organ systems involved by disease outside of the organ system of the treatment target, per prior convention in the literature (15, 16).

### Risk score validation

**Figure 2:**
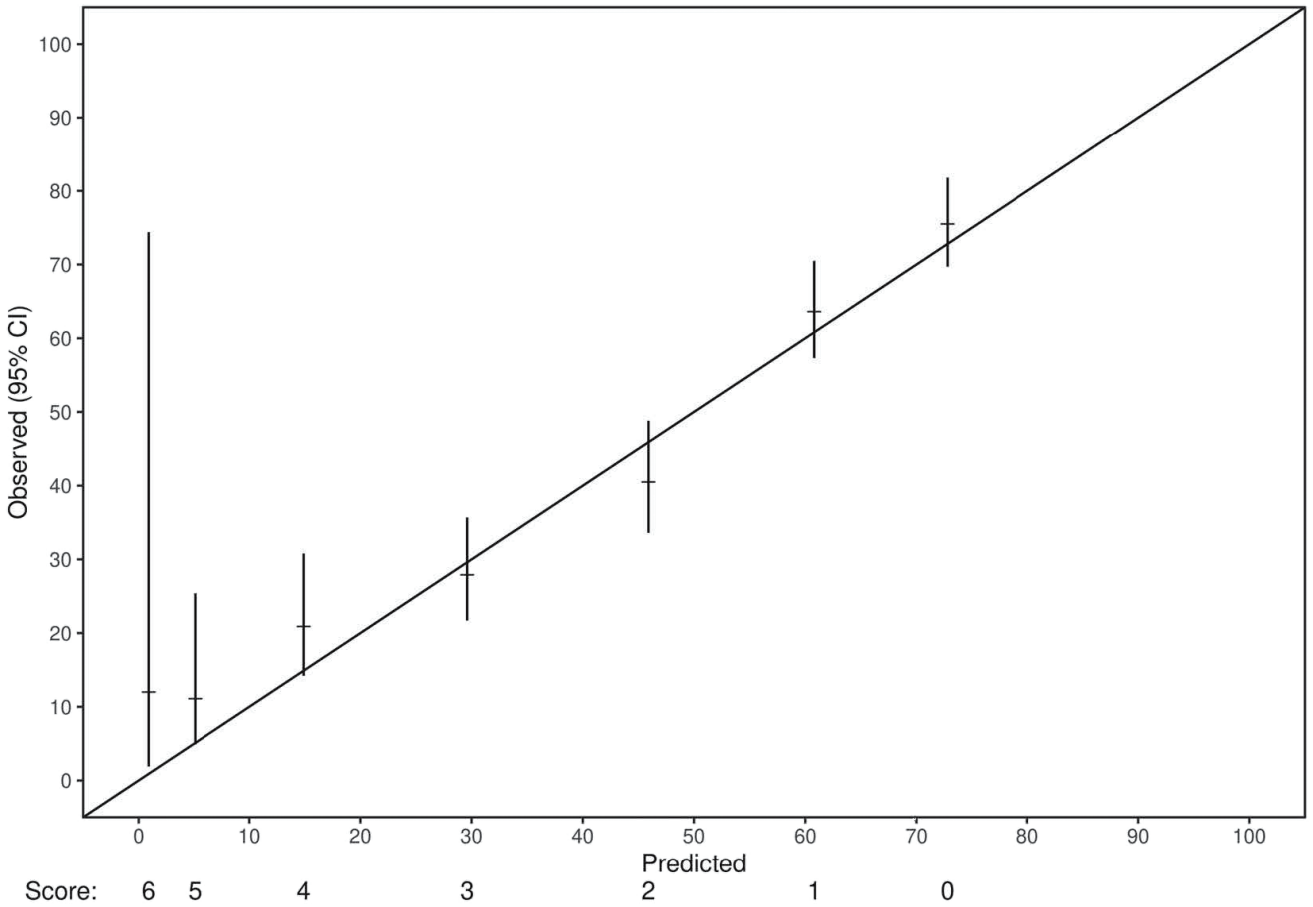
A demonstration of the adequacy of the validation set for each score of the final prognostic model. For all scores but one, the number of events in the validation set fit within the 95% confidence interval of the predicted event frequencies. The only exception was the score of 6, which only occurred 11 times in the training set and was, therefore, only a very small fraction of the final prognostic score.

Cox proportional hazards analysis revealed a concordance value of 0.73 (95% CI: 0.71-0.75) for the risk score in predicting OS in the training set. Similarly, the risk score had a concordance value of 0.72 (95% CI: 0.68-0.74) for predicting OS in the internal validation set. Each individual variable within the risk score also demonstrated statistical significance in both the training and internal validation sets (p<0.0001) (**Table 2**). Each stratification of these variables also revealed a statistically significant association with OS (p ≤ 0.01). Next, the applicability of the internal validation set for each individual score of the resulting risk score was assessed (**Figure 2**). The observed OS for each risk group was within the 95% confidence interval of the predicted OS, except for the small group of score 6. The score of 6 only occurred 11 times in the training set and was, therefore, only a very small fraction of the final prognostic score.

### Treatment target analyses

The resulting risk score was next individually applied to each of the 3 treatment targets involved in the overall cohort. Concordance values were 0.72 (95% CI: 0.68-0.74), 0.71 (95% CI: 0.68-0.75), and 0.65 (95% CI: 0.62-0.68) for lung, spine, and brain, respectively. Considering patients with intracranial targets, statistical significance was maintained when considering patients with only a single brain metastasis (n=150, p=0.0003) or for patients with multiple brain metastases (n=274, p<0.0001). Both ECOG and NOS demonstrated statistical significance in each subset (p<0.0001) (**Supplemental Table 3**). Comparable hazard ratios were identified in the 3 subsets for each stratification of ECOG and NOS. Each stratification of these variables also revealed a statistically significant association with OS, except for NOS = 1 for the lung cohort (HR=1.31, 95% CI: 0.89-1.94, p=0.17). Similarly, the model demonstrated prognostic value for each of the most common primary histologies (breast, lung, and GI), and each individual variable within the risk score demonstrated a statistically significant association with OS within each primary histology (**Supplemental Table 4**).

## Discussion

Using a large, multi-institutional cohort, we developed and validated a pre-treatment prognostic risk score for OS to aid in selection of patients most likely to benefit from metastasis-directed radiotherapy. Recent advances in systemic therapy have substantially improved OS for patients with metastatic disease, allowing additional opportunities to incorporate MDT for patients (31, 32). Therefore, our model provides a tool to identify patients who may derive the most benefit from MDT, and it also identifies those who may be spared MDT and the associated risks of toxicity (4, 8). Unique to our study is the inclusion of both intracranial and extracranial targets, and our resulting concordance index was higher in both the training and validation sets than prior models assessing only extracranial disease (15, 18). The inclusion of patients with brain metastasis adds critical data to the topic of patient selection for MDT reflective of true clinical practice, as approximately a quarter of patients who die of cancer develop brain metastasis (33, 34). Therefore, our model may be useful to guide future MDT clinical trial inclusion criteria design, as well as to provide a unifying model for patients with intracranial and extracranial disease.

Quantification of a patient’s overall metastatic disease burden is a useful tool to guide clinical decision making for MDT and characterize a patient’s prognosis. One clear example is in the setting of prostate cancer, where clinical trials suggest that the role of radiotherapy differs in patients with low volume vs. high volume metastatic disease (35). The optimal manner to stratify other metastatic patients, however, remains unclear. Past approaches include reporting the number of total metastatic lesions, total volume of metastatic lesions, or number of organ systems involved by metastatic disease (36). Other data suggest that the number of metastatic lesions alone is an incomplete prognostic variable, and the number of non-target organ systems involved by disease or the presence of only a solitary metastasis may be superior prognostic variables (15, 16). For example, the classification from the CHAARTED trial included two criteria for high volume metastatic burden: 4 or more bone metastases with one or more outside the vertebral bodies or pelvis, or visceral metastases (or both) (37). That is, five metastases within vertebral bodies would only classify as low metastatic burden, just as our model would have accounted for this scenario with an NOS of 0. Alternatively, the presence of a single vertebral body lesion and a single visceral metastasis would have qualified as high metastatic burden, and our model would have incorporated an NOS of 1. Stratification by only the number of metastatic lesions, however, would have allowed the latter patient to enroll in many oligometastatic clinical trials due to the presence of only 2 sites of metastatic disease. This difference may allow for more accurate prognostication using our model and NOS, in line with the CHAARTED definition.

An additional concern with stratification by only the number of metastatic lesions is that as imaging sensitivity and sophistication increases, the number of previously occult metastatic lesions identified are likely to increase, making comparison and applicability of prior studies using this classification challenging (19). Therefore, prior analyses would have underestimated the true number of metastatic sites, and it remains unknown whether the presence of additional, small, clinically occult lesions within the same organ system substantially impacts patient prognosis.

Perhaps the most unique aspect of our study was the inclusion of intracranial targets. A substantial fraction (34%) of these patients were treated with WBRT, which involves treating the entire brain to cover subclinical disease. Furthermore, 75% of patients treated for intracranial disease also had extracranial disease, and the model still effectively stratified such patients by OS. This result suggests that patients with intracranial metastatic disease and otherwise favorable characteristics (e.g. ECOG of 0-1 and NOS of 0-1) could still be candidates for MDT, despite a larger number of individual metastatic lesions than included in most clinical trials. Therefore, we propose that carefully selected patients with intracranial metastatic disease be further studied to consider potential inclusion in future clinical trials (20).

ECOG performance status was identified as an important prognostic variable in our analysis, consistent with prior studies (17). In the setting of brain metastases, rigorous graded prognostic assessment has consistently identified performance status as a crucial prognostic variable across multiple primary tumor histologies (20). Multiple prognostic systems have been constructed for spine SBRT incorporating performance status as a key variable (15, 16, 38, 39). Systematic reviews have noted encouraging inter-rater reliability for performance status scoring, with no clear superiority of one system over the other (40, 41). Overall, we strongly encourage utilization of performance status to aid in prognostication of OS and ultimately patient selection for MDT.

A limitation of our analysis is that the model was developed using retrospective data. However, the large size of our patient cohort (1,260 patients) and multi-institutional involvement, are substantial strengths of our analysis. Patients were also treated with modern imaging and radiotherapy treatment techniques, allowing us to adequately capture current clinical practice. Next, while we did not directly test the number of metastatic lesions as a potential prognostic variable, the number of metastatic lesions would have demonstrated strong correlation with NOS. We acknowledge that clinical judgement will be required to incorporate this model in a given clinical context. Finally, this model is most generalizable to patients with GI, breast, and NSCLC primary tumors, which constituted the histologies of patients with intracranial metastases included in this study. Even so, patients with spine SBRT and lung SBRT had a range of primary histologies, and primary tumor histology was not found to be a key prognostic variable in that setting (42). Therefore, the authors feel that it would be appropriate to generalize this model outside of only primary GI, breast, and NSCLC.

## Conclusions

Our pre-treatment decision tool identifies patients most likely to benefit from metastasis-directed radiotherapy secondary to favorable OS and applies for both intracranial and extracranial disease. We encourage future clinical trial design to consider ECOG and NOS as prognostic variables for patients with metastatic disease. Carefully selected patients may benefit from MDT even in the presence of intracranial disease.

## Data availability statement

The original contributions presented in the study are included in the article/**Supplementary Material**. Further inquiries can be directed to the corresponding author.

## Ethics statement

The studies involving human participants were reviewed and approved by Mayo Clinic Institutional Review Board. Written informed consent for participation was not required for this study in accordance with the national legislation and the institutional requirements.

## Author contributions

All authors listed have made a substantial, direct, and intellectual contribution to the work and approved it for publication.
