# Supplementary material for: Development and validation of a unifying pre-treatment decision tool for intracranial and extracranial metastasis-directed radiotherapy
Source: Front Oncol. 2023 Mar 27;13:1095170. doi: 10.3389/fonc.2023.1095170 (PMC10083422; doi:10.3389/fonc.2023.1095170)
Supplement: Supplementary file 1 [file Table_1.docx]

**Supplemental tables**

**Supplemental table 1**. Further details concerning the patient details of patients treated for each of the target organ systems investigated are tabulated.

|  | **Spine** | **Lung** | **Intracranial** |
| --- | --- | --- | --- |
| *Patients*  Female  Male | 606  216 (36%)  390 (64%) | 352  174 (49%)  178 (51%) | 424  300 (71%)  124 (29%) |
| *Treatment dates* | 11/2002 – 10/2019 | 05/2013 – 12/2020 | 04/2004 – 12/2020 |
| *Median age (years (IQR))* | 63 (55-71) | 65 (55-73) | 64 (55-72) |
| *Most common primary histologies* | Prostate (24%)  Renal (23%)  NSCLC (11%) | Melanoma (19%)  Colorectal (16%)  Sarcoma (9%)  Renal (9%) | Breast (44%)  NSCLC (36%)  GI (20%) |

Abbreviations: Dates are abbreviated as mm/yyyy; IQR = interquartile range; NSCLC = non-small cell lung cancer; GI = gastrointestinal

**Supplemental table 2**. Additional characteristics of patients treated with brain metastases are described.

| **Characteristic** | **Incidence** |
| --- | --- |
| *Patients*  Female  Male | 300 (71%)  124 (29%) |
| *Primary tumor*  Breast  NSCLC  GI | 186 (44%)  153 (36%)  85 (20%) |
| *Number of brain metastases*  > 10  Median* | 78 (18%)  2 (IQR: 1-3) |
| *Treatment modality*  SRS  WBRT | 349 (82%)  145 (34%) |
| *Extracranial disease burden*  No other organ systems involved  Liver metastases  Bone metastases  Lung metastases  Other metastases | 107 (25%)  112 (26%)  182 (43%)  162 (38%)  174 (41%) |

*The median number is provided for all patients who had 10 or fewer metastatic lesions

Abbreviations: NSCLC = non-small cell lung cancer; GI = gastrointestinal; IQR = interquartile range; SRS = stereotactic radiosurgery; WBRT = whole brain radiation therapy

**Supplemental table 3**. The model showed prognostic values across each treatment target considered in the analysis. Concordance values were 0.72 (95% confidence interval (CI): 0.68-0.74), 0.71 (95% CI: 0.68-0.75), and 0.65 (0.62-0.68) for lung, spine, and brain, respectively. In all instances except for 1 additional organ system in the lung SBRT cohort, all model components demonstrated statistically significant prognostic power within each treatment target subset.

| **Model component** | **Lung (hazard ratio, 95% CI)** | **Spine (hazard ratio, 95% CI)** | **Brain (hazard ratio, 95% CI)** |
| --- | --- | --- | --- |
| *ECOG*  0  1  > 1 | Overall: p<0.0001  1.0 (reference)  1.73 (1.25-2.38), p=0.0009  6.46 (4.14-10.09), p<0.0001 | Overall: p<0.0001  1.0 (reference)  2.31 (1.75-3.04), p<0.0001  6.43 (4.42-9.35), p<0.0001 | Overall: p<0.0001  1.0 (reference)  1.85 (1.45-2.37), p<0.0001  2.91 (1.97-4.29), p<0.0001 |
| *NOS*  0  1  > 1 | Overall: p<0.0001  1.0 (reference)  1.31 (0.89-1.94), p=0.17  4.17 (2.43-7.14), p<0.0001 | Overall: p<0.0001  1.0 (reference)  1.36 (1.00-1.84), p=0.048  2.28 (1.67-3.11), p<0.0001 | Overall: p<0.0001  1.0 (reference)  1.56 (1.15-2.13), p=0.0045  2.09 (1.57-2.78), p<0.0001 |

Abbreviations: CI = confidence interval; ECOG = Eastern Cooperative Oncology Group performance status; NOS = number of organ systems involved (outside of treatment target)

**Supplemental table 4**. The applicability of the model to each specific treatment target is shown. Concordance values were 0.62 (95% confidence interval (CI): 0.58-0.66), 0.72 (95% CI: 0.67-0.76), and 0.68 (0.63-0.72) for breast, lung, and gastrointestinal (GI), respectively. Each variable demonstrating within each primary histology demonstrated a statistically significant association with overall survival.

| **Model component** | **Breast (hazard ratio, 95% CI), n=278** | **Lung (hazard ratio, 95% CI), n=150 patients** | **GI (hazard ratio, 95% CI), n=150 patients** |
| --- | --- | --- | --- |
| *ECOG*  0  1  > 1 | Overall: p=0.023  1.0 (reference)  1.11 (0.81-1.52), p=0.53  1.67 (1.16-2.41), p=0.0063 | Overall: p=0.002  1.0 (reference)  2.01 (1.42-2.87), p<0.0001  0.98 (0.54-1.76), p=0.94 | Overall: p=0.0049  1.0 (reference)  1.33 (0.82-2.16), p=0.25  2.48 (1.43-4.30), p=0.0012 |
| *NOS*  0  1  > 1 | Overall: p<0.0001  1.0 (reference)  1.48 (1.0-2.20), p=0.052  2.38 (1.68-3.35), p<0.001 | Overall: p<0.0001  1.0 (reference)  1.66 (1.12-2.45), p=0.011  3.75 (2.40-5.87), p<0.0001 | Overall: p<0.0001  1.0 (reference)  2.38 (1.47-3.86), p=0.0005  6.41 (3.87-10.62), p<0.0001 |

Abbreviations: CI = confidence interval; ECOG = Eastern Cooperative Oncology Group performance status; NOS = number of organ systems involved (outside of treatment target)
